# Melatonin Protects against Apoptosis-Inducing Factor (AIF)-Dependent Cell Death during Acetaminophen-Induced Acute Liver Failure

**DOI:** 10.1371/journal.pone.0051911

**Published:** 2012-12-18

**Authors:** Ying-Li Liang, Zhi-Hui Zhang, Xiao-Jing Liu, Xiao-Qian Liu, Li Tao, Ye-Fa Zhang, Hua Wang, Cheng Zhang, Xi Chen, De-Xiang Xu

**Affiliations:** 1 Department of Toxicology, Anhui Medical University, Hefei, China; 2 First Affiliated Hospital, Anhui Medical University, Hefei, China; Faculté de médecine de Nantes, France

## Abstract

Acetaminophen (APAP) overdose is the most frequent cause of acute liver failure and is primarily caused by cytochrome P450 (CYP) 2E1-driven conversion of APAP into hepatotoxic metabolites. Several reports showed that melatonin attenuated APAP-induced acute liver failure. Nevertheless, the exact mechanism remains obscure. In the present study, we investigated the effects of melatonin on apoptosis-inducing factor (AIF)-dependent cell death in APAP-induced acute liver failure. Mice were intraperitoneally (i.p.) injected with different doses of melatonin (1.25, 5, 20 mg/kg) 30 min before APAP (300 mg/kg, i.p.). As expected, melatonin significantly alleviated APAP-induced cell death, as determined by TdT-mediated dUTP-biotin nick end labeling (TUNEL) assay. Further analysis showed that melatonin significantly attenuated APAP-induced activation of the serine/threonine kinase receptor interacting protein 1 (RIP1). In addition, melatonin inhibited APAP-induced hepatic c-Jun N-terminal kinase (JNK) phosphorylation and mitochondrial Bax translocation. Correspondingly, melatonin inhibited APAP-induced translocation of AIF from mitochondria to nuclei. Interestingly, no changes were induced by melatonin on hepatic CYP2E1 expression. In addition, melatonin had little effect on APAP-induced hepatic glutathione (GSH) depletion. In conclusion, melatonin protects against AIF-dependent cell death during APAP-induced acute liver failure through its direct inhibition of hepatic RIP1 and subsequent JNK phosphorylation and mitochondrial Bax translocation.

## Introduction

Acetaminophen (APAP) is a widely used analgesic and antipyretic drug. Although safe at therapeutic doses, APAP overdose can cause severe acute liver damage characterized by centrilobular hepatic necrosis [1]. APAP-induced hepatotoxicity is initiated by the formation of a reactive metabolite, N-acetyl-p-benzoquinone imine (NAPQI), which can be generated by several hepatic cytochrome P-450 (CYP) isoenzymes, especially CYP2E1 [2]. Increasing evidence demonstrates that apoptosis-inducing factor (AIF), which translocates to the nucleus and initiates nuclear DNA fragmentation, may be a critical mediator of APAP-induced cell death [3]–[5]. The prolonged activation of c-Jun N-terminal kinase (JNK) plays a key role in APAP-induced cell death [6]. An earlier report showed that leflunomide, antirheumatic drug, protected mice from APAP-induced acute liver damage through inhibition of JNK phosphorylation [7]. A recent study showed that arjunolic acid, a triterpenoid saponin, prevented from APAP-induced acute liver failure through inhibiting JNK-mediated activation of mitochondrial permeabilization [8].

Melatonin is the major secretory product of the pineal gland. As a potent antioxidant, melatonin and its metabolites directly scavenge a variety of free radicals [9]–[12]. Moreover, melatonin exhibits its indirect antioxidant role through stimulating superoxide dismutase (SOD) and glutathione peroxidase (GSH-Px) activities and upregulating the expression of antioxidant enzymes [13]–[18]. Melatonin has an anti-apoptotic effect [19], [20]. According to an earlier report, melatonin protected mice from lipopolysaccharide/D-galactosamine-induced apoptotic liver damage [21]. In addition, pretreatment with melatonin prevents from ischemia/reperfusion-induced hepatic necrosis and apoptosis [22], [23]. Several reports showed that melatonin attenuated APAP-induced acute liver failure [24], [25]. Nevertheless, the exact mechanism remains obscure.

In the present study, we investigated the effects of melatonin on AIF-dependent cell death in a mouse model of APAP-induced acute liver failure. We demonstrate for the first time that melatonin protects against AIF-dependent cell death during APAP-induced acute liver failure through its direct inhibition of hepatic receptor interacting protein 1 (RIP1) activation and subsequent JNK phosphorylation and mitochondrial Bax translocation.

## Materials and Methods

### Chemicals and Reagents

Acetaminophen (APAP) and melatonin were purchased from Sigma Chemical Co. (St. Louis, MO). Antibodies against RIP1, phosphor-JNK (pJNK), Bcl-2, Bax, AIF and cytochrome c were from Santa Cruz Biotechnologies (Santa Cruz, CA). Porin antibody was from Abcam Ltd., (Cambridge, UK). β-actin antibody was from Boster Bio-Technology Co. LTD (Wuhan, China). Chemiluminescence (ECL) detection kit was from Pierce Biotechnology (Rockford, IL). All the other reagents were from Sigma or as indicated in the specified methods.

### Animals and Treatments

Male CD-1 mice (6∼8 week-old, 22∼24 g) were purchased from Beijing Vital River (Beijing, China). The animals were allowed free access to food and water at all times and were maintained on a 12-h light/dark cycle in a controlled temperature (20–25°C) and humidity (50±5%) environment for a period of 1 week before use. Thirty-six mice were divided into six groups. After a 12-h fast, all mice except controls were intraperitoneally (i.p.) injected with APAP (300 mg/kg). In APAP+melatonin groups, mice were i.p. injected with different doses of melatonin (1.25, 5, 20 mg/kg) 30 min before APAP (300 mg/kg, i.p.). This study was approved by the Association of Laboratory Animal Sciences and the Center for Laboratory Animal Sciences at Anhui Medical University (Permit Number: 11-0012). All procedures on animals followed the guidelines for humane treatment set by the Association of Laboratory Animal Sciences and the Center for Laboratory Animal Sciences at Anhui Medical University.

### Biochemical Parameters and Histology

The levels of serum alanine aminotransferase (ALT) were measured using commercially available assay kits according to the manufacturer’s instructions. Liver tissues were fixed in 4% formalin and embedded in paraffin according to the standard procedure. Paraffin embedded tissues were cut 5 µm thick and stained with hematoxylin and eosin (H & E) for morphological analysis.

### Subcellular Fractionation

Cytosolic and mitochondrial fractions were prepared as described previously [26], [27]. Briefly, liver tissues were homogenized using a dounce homogenizer in 3 ml buffer A (0.25 M sucrose, 50 mM Hepes, 10 mM NaCl, 10 mM EDTA, 2 mM dithiothreitol) supplemented with protease inhibitors (Complete Protease Inhibitors; Roche, Indianapolis, IN). The crude homogenates were centrifuged at 1000×*g* for 10 min at 4°C and the resultant supernatant centrifuged at 10 000×*g* for 15 min at 4°C to sediment the low-speed fraction containing mainly mitochondria. The mitochondria were washed two times in buffer A and pelleted. The cytosolic and high-speed fractions were isolated following centrifugation of the 10 000×*g* supernatant fraction at 100 000×*g* for 60 min at 4°C. The resulting supernatant was the cytosolic fraction.

### Immunoblots

Immunoblots were performed using liver lysates and subcellular fractions. In brief, protein extracts from each sample were separated electrophoretically by SDS-PAGE and transferred to a polyvinylidene fluoride membrane. For mitochondrial protein, the membranes were incubated for 2 h with following antibodies: Bax and Bcl-2. For cytosolic protein, the membranes were incubated for 2 h with Cyt c antibody. For total proteins, the membranes were incubated for 2 h with following antibodies: RIP1, p-JNK and CYP2E1. For total proteins and cytosolic protein, β-actin was used as a loading control. For mitochondrial protein, Porin was used as a loading control. After washes in DPBS containing 0.05% Tween-20 four times for 10 min each, the membranes were incubated with goat anti–rabbit IgG antibody for 2 h. The membranes were washed for four times in DPBS containing 0.05% Tween-20 for 10 min each, followed by signal development using an ECL detection kit.

### Immunohistochemistry

Paraffin-embedded sections were deparaffinized and rehydrated in a graded ethanol series. After antigen retrieval and quenching of endogenous peroxidase, sections were incubated with AIF monoclonal antibodies (1∶200 dilution) at 4°C overnight. The color reaction was developed with HRP-linked polymer detection system and counterstaining with hematoxylin.

### Terminal dUTP Nick-end Labeling (TUNEL) Assay

For the detection of nuclear DNA strand breaks, paraffin-embedded sections were stained with the TUNEL technique using an in situ apoptosis detection kit (Promega) according to the manufacturer’s protocols. Sections were counterstained with hematoxylin. TUNEL-positive cells were counted in twelve randomly selected fields from each slide at a magnification of ×200. The percentage of TUNEL-positive cells was analyzed in six liver sections from six different mice.

### Isolation of Total RNA and Real-time RT-PCR

Total RNA was extracted using TRI reagent according to the manufacturer’s instructions. The purity of RNA was assessed according to the ratio of absorbance at 260 nm and 280 nm. RNase-free DNase (Promega, Madison, WI, USA) was used to remove genomic DNA. The cDNA was synthesized from 2 µg of total RNA using Avian Myeloblastosis Virus (AMV) reverse transcriptase (Pregmega, Madison, WI, USA). Real-time RT-PCR was performed with a LightCycler® 480 SYBR Green I kit (Roche Diagnostics GmbH, Mannheim, Germany) using gene-specific primers. PCR for glyceraldehyde-3-phosphate dehydrogenase (GAPDH) was performed on each individual sample as an internal positive-control standard. The primers were synthesized by Shanghai Sangon Biological Engineering Technology and Service Company (Shanghai, China) as listed in Table 1. The comparative C_T_-method was used to determine the amount of target, normalized to an endogenous reference (GAPDH) and relative to a calibrator (2^−ΔΔCt^) using the Lightcycler 480 software (Roche, version 1.5.0). The purity of PCR products was verified by melting curves. All RT-PCR experiments were performed in triplicate.

**Table 1:** Primers for real-time RT-PCR.

Genes	Sequences	Sizes (bp)
*Gapdh*	Forward: 5′- ACCCCAGCAAGGACACTGAGCAAG -3′	109
	Reverse: 5′- GGCCCCTCCTGTTATTATGGGGGT -3′	
*Cyp2e1*	Forward: 5′- CCGACCTGTTCTTTGCAGGA -3′	128
	Reverse: 5′- GCTTGGCCCAATAACCCTGT -3′	
*GSH-Px1*	Forward: 5′- GGTGGTGCTCGGTTTCCCGT -3′	113
	Reverse: 5′- AATTGGGCTCGAACCCGCCAC -3′	
*GSH-Rd*	Forward: 5′- GGGATGCCTATGTGAGCCGCC -3′	120
	Reverse: 5′- TGACTTCCACCGTGGGCCGA -3′	
*SOD1*	Forward: 5′- GCGATGAAAGCGGTGTGCGTG -3′	143
	Reverse: 5′- TGGACGTGGAACCCATGCTGG -3′	
*Catalase*	Forward: 5′- CGCGCTCGAGTGGCCAACT -3′	107
	Reverse: 5′- TGCTGCTCTGGTGCGCTGAA -3′	

### Determination of Glutathione Content

The GSH and GSSG contents of liver were determined by the DTNB–GSSG reductase recycling assay as described by Anderson [28], with some modifications. Briefly, 0.4 ml liver homogenates were precipitated by the addition of 0.4 ml of a metaphosphoric acid solution and centrifuged for 10 min at 13,000 rpm in a refrigerated centrifuge (4°C). After this time, 0.1 ml acidic supernatant was neutralized with 0.1 ml 0.76 M KHCO_3_ and the sample centrifuged for 1 min at 13,000 rpm. Fresh reagent, containing 0.69 mM NADPH and 4 mM DTNB in 72 mM phosphate buffer, was prepared daily. For measurement of total glutathione, 100 µl/well of samples, standards or blank well were added in duplicate to 96-well microtiter plates, followed by 65 µl/well of the freshly prepared reagent. Plates were then incubated in a thermomax plate reader (PowerWaveX; Bio-Tek, Winooski, VT, USA) at 30°C for 10 min prior to the addition of 40 µl GR per well (10 IU/ml in phosphate buffer). The stoichmetric formation of 5-thio-2-nitrobenzoic acid (TNB) was followed for 2 min at 415 nm and compared with a standard curve. For the determination of GSSG, aliquots (200 µl) of acidic supernatant were treated with 10 µl 2-vinylpyridine and mixed continuously for 1 h for derivati-zation of GSH. GSSG was then measured as described above for total glutathione. The GSH levels were calculated by subtracting GSSG content from the total glutathione content (GSH = GSH_t_−2×GSSG).

### Statistical Analysis

All data were expressed as means±SEM at each point. ANOVA and the Student-Newmann-Keuls post hoc test were used to determine differences among different groups. Differences were considered significant only for *P*<0.05.

## Results

**Figure 1:**
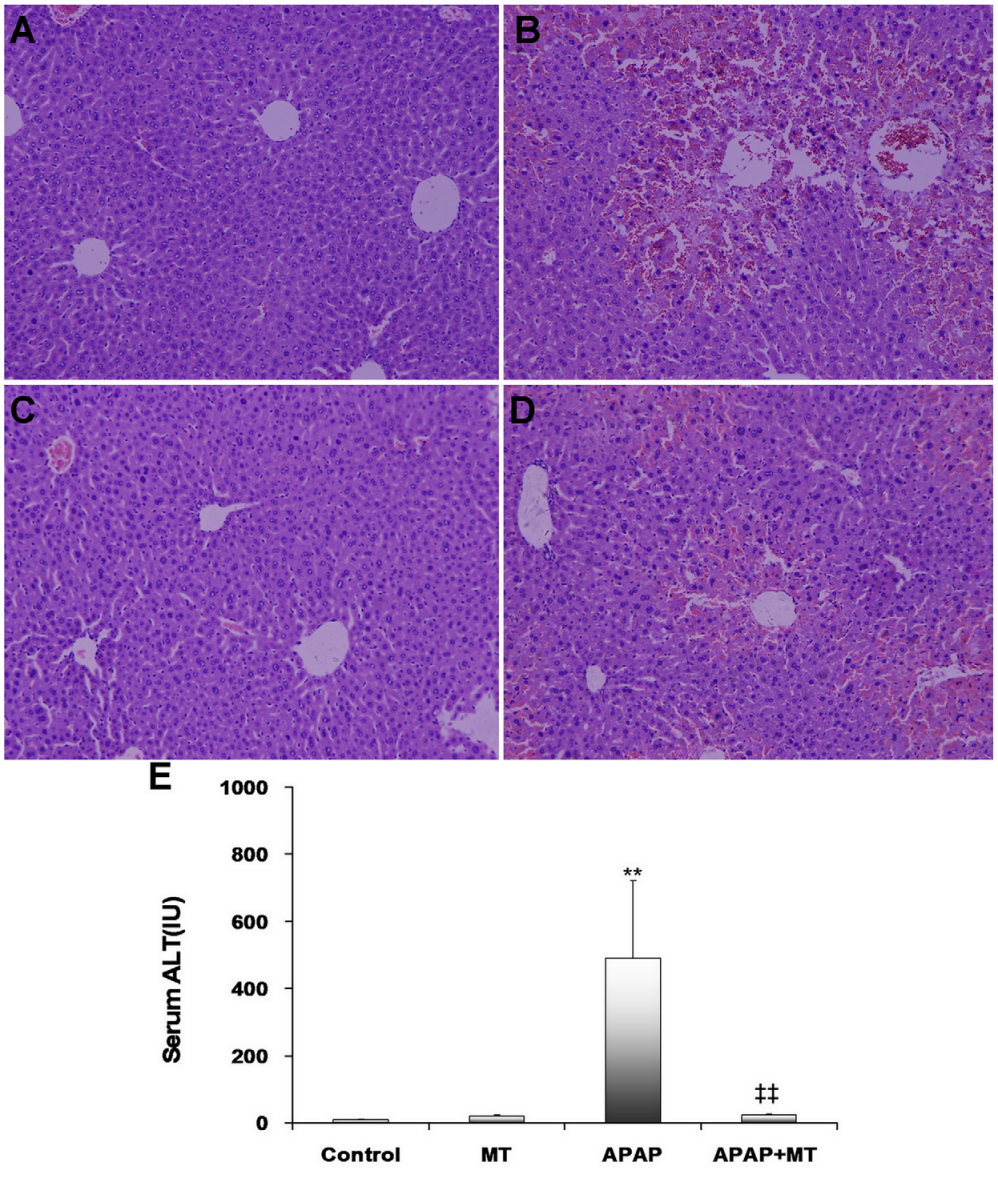
Melatonin attenuates APAP-induced acute liver injury. Mice were treated as Materials and Methods. Liver samples were collected at 4 h after APAP administration. Representative photomicrographs of liver histology from mice treated with saline (A as control), APAP alone (B), melatonin alone (C), and combination of APAP and melatonin (D) are shown (H & E, magnification: 100×). (E) Sera were collected at 4 h after APAP administration. Serum ALT was measured. All data were expressed as means ± SEM (n = 6). **P<0.01 as compared with the control. ‡‡ *P*<0.01 as compared with APAP group.

**Figure 2:**
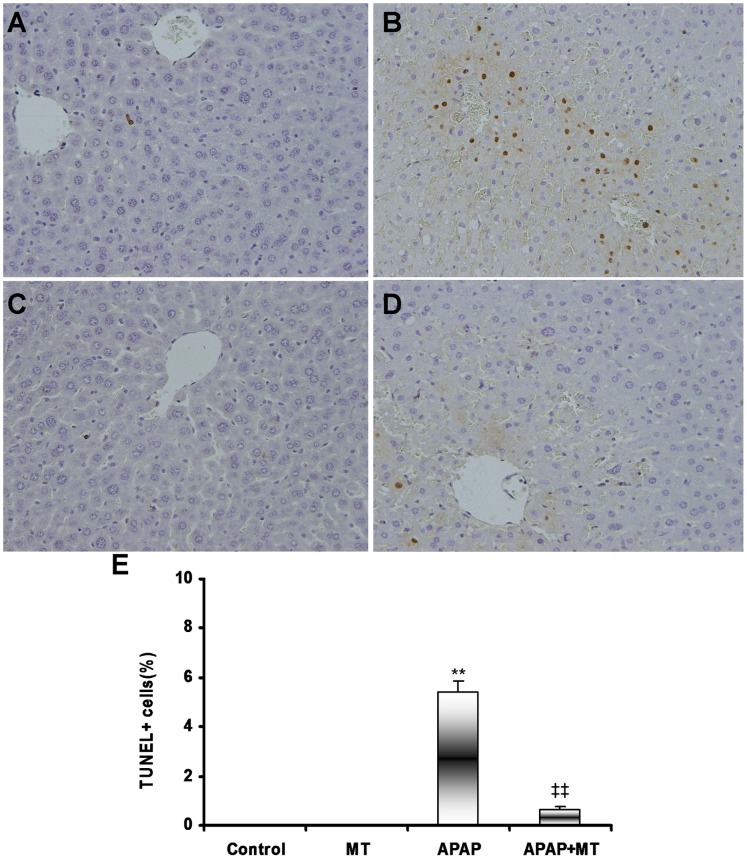
Melatonin protects against APAP-induced hepatocyte death. Mice were treated as Materials and Methods. Liver samples were collected at 4 h after APAP administration. Hepatocyte death was determined using TUNEL assay. Representative photomicrographs of liver section from mice treated with saline (A as control), APAP alone (B), melatonin alone (C), and combination of APAP and melatonin (D) are shown. (E) TUNEL+ cells were analyzed. All data were expressed as means ± SEM (n = 6). **P<0.01 as compared with the control. ‡‡ *P*<0.01 as compared with APAP group.

As shown in Fig. 1B, hepatic histopathology showed an obvious congestion in liver section of mice administered with APAP. Pretreatment with melatonin significantly alleviated APAP-induced hepatic congestion (Fig. 1D). Further analysis showed that pretreatment with melatonin significantly attenuated APAP-induced elevation of serum ALT (Fig. 1E). APAP-induced cell death was determined by TUNEL assay. As shown in Fig. 2B, numerous TUNEL+ cells were observed in liver of mice administered with APAP. Interestingly, melatonin significantly attenuated APAP-induced increase in hepatic TUNEL+ cells (Fig. 2E).

**Figure 3:**
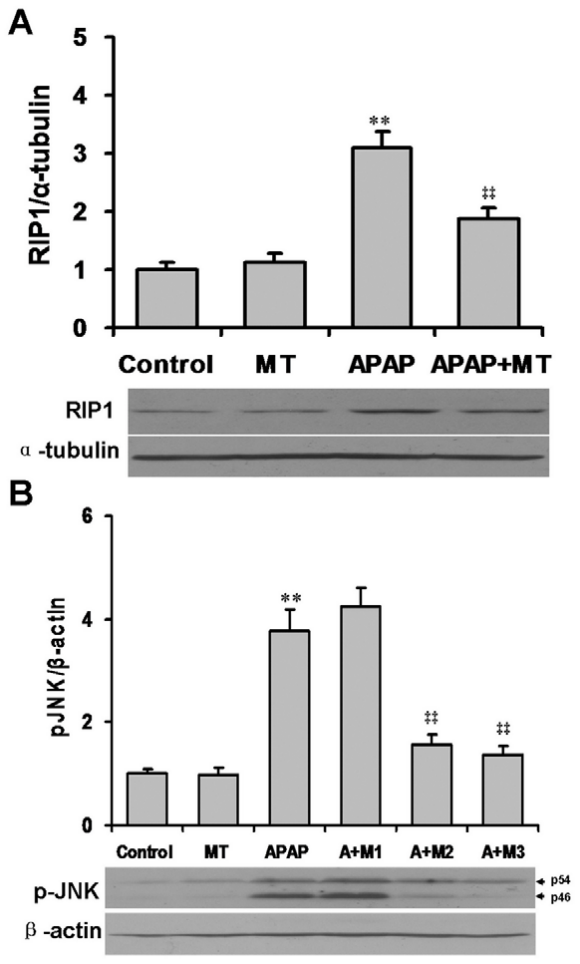
Melatonin attenuates APAP-induced hepatic RIP1 activation and JNK phosphorylation. Mice were treated as Materials and Methods. Liver samples were collected at 1 h after APAP administration. (A) Hepatic RIP1 was detected by immunoblots. (B) All mice except controls were i.p. injected with APAP (300 mg/kg). In melatonin+APAP group, mice were i.p. injected with different doses of melatonin (1.25, 5, 20 mg/kg) 30 min before APAP (300 mg/kg, i.p.). Liver samples were collected at 4 h after APAP administration. Hepatic phosphorylated JNK was detected by immunoblots. All experiments were repeated for four times. Quantitative analyses of scanning densitometry on four different samples were performed. All data were expressed as means±SEM (n = 4). **P<0.01 as compared with the control. ‡‡ *P*<0.01 as compared with APAP group.

The effects of melatonin on APAP-induced expression of hepatic RIP1 were analyzed. As shown in Fig. 3A, the level of hepatic RIP1 was significantly increased in APAP-treated mice. Pretreatment with melatonin significantly attenuated APAP-induced upregulation of hepatic RIP1. The effects of melatonin on APAP-induced hepatic JNK phosphorylation are presented in Fig. 3B. As expected, the level of phosphorylated JNK was significantly increased in liver of mice administered with APAP. Interestingly, pretreatment with melatonin inhibited APAP-evoked hepatic JNK phosphorylation in a dose-dependent manner.

**Figure 4:**
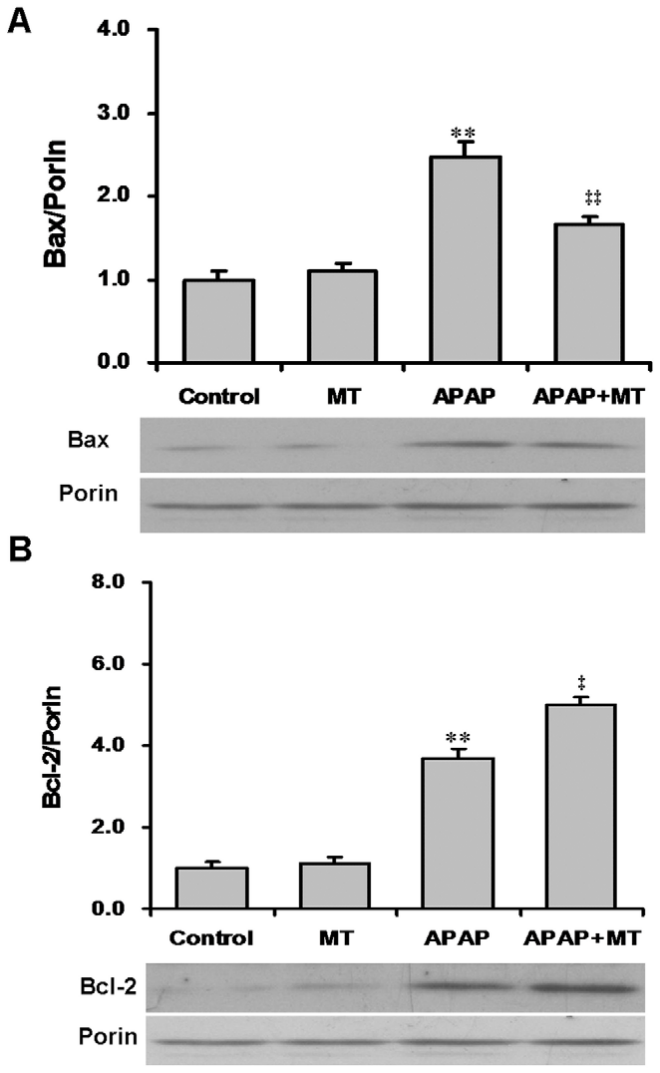
Effects of melatonin on APAP-induced mitochondrial Bax and Bcl-2 translocation. Mice were treated as Materials and Methods. Liver samples were collected at 4 h after APAP. (A) Bax and (B) Bcl-2 in mitochondria were detected by immunoblots. All experiments were repeated for four times. Quantitative analyses of scanning densitometry on four different samples were performed. All data were expressed as means±SEM (n = 4). **P<0.01 as compared with the control. ‡ *P*<0.05, ‡‡ *P*<0.01 as compared with APAP group.

The effects of melatonin on APAP-induced translocation of Bax are shown in Fig. 4A. As expected, the level of mitochondrial Bax was significantly increased in liver of mice administered with APAP. Interestingly, melatonin significantly attenuated APAP-induced translocation of Bax from the cytosol to the mitochondria (Fig. 4A). The effects of melatonin on APAP-induced translocation of Bcl-2 were then analyzed. As shown in Fig. 4B, the level of mitochondrial Bcl-2 was significantly increased in liver of mice administered with APAP. Unexpectedly, pretreatment with melatonin aggravated the level of mitochondrial Bcl-2 in liver of mice administered with APAP (Fig. 4B).

**Figure 5:**
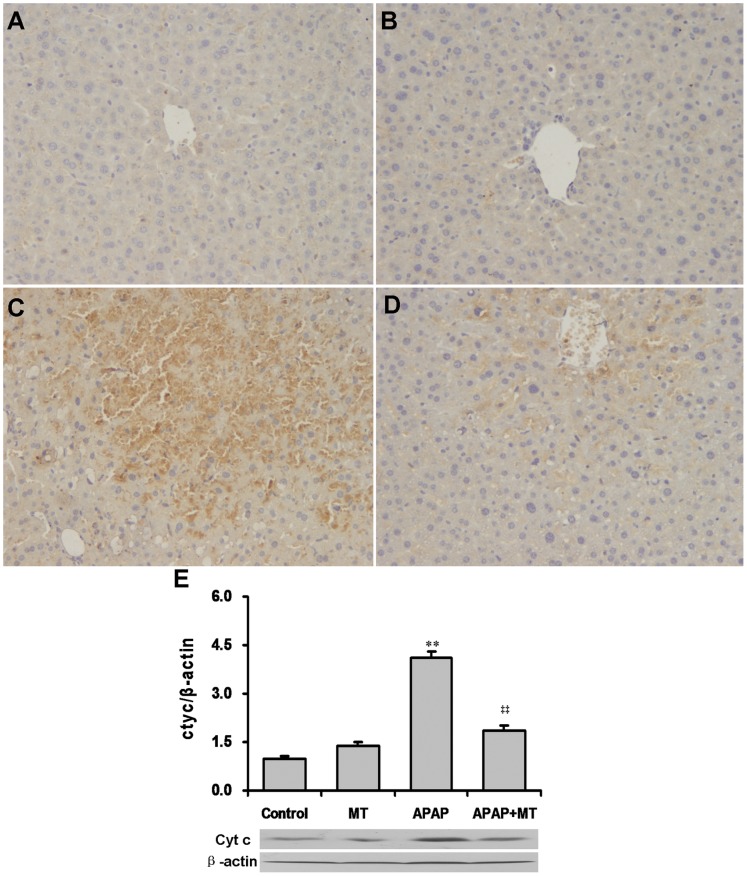
Effects of melatonin on APAP-induced release of cytochromec and AIF translocation. Mice were treated as Materials and Methods. Liver samples were collected at 4 h after APAP administration. Nuclear translocation of AIF was analyzed using immunohistochemistry. Representative photomicrographs of liver histology from mice treated with saline (A as control), melatonin alone (B), APAP alone (C) and melatonin+APAP (D) are shown. Original magnification: 200×.(E) Cyt c in cytosol was detected by immunoblots. All experiments were repeated for four times. All data were expressed as means ± SEM (n = 4). **P<0.01 as compared with the control. ‡‡ *P*<0.01 as compared with APAP group.

The effects of melatonin on APAP-induced nuclear translocation of AIF were then analyzed. Immunohistochemistry showed that nuclear translocation of AIF was mainly distributed around hepatic sinus, where nuclei were partially digested (Fig. 5C). Interestingly, pretreatment with melatonin significantly attenuated APAP-induced hepatic nuclear AIF translocation (Fig. 5D). The effects of melatonin on APAP-induced release of hepatic cyt c are shown in Fig. 5E. As expected, the level of cyt c in the cytosol was significantly increased in liver of mice administered with APAP. Interestingly, pretreatment with melatonin significantly attenuated APAP-induced release of cyt c from the mitochondria to the cytosol.

**Figure 6:**
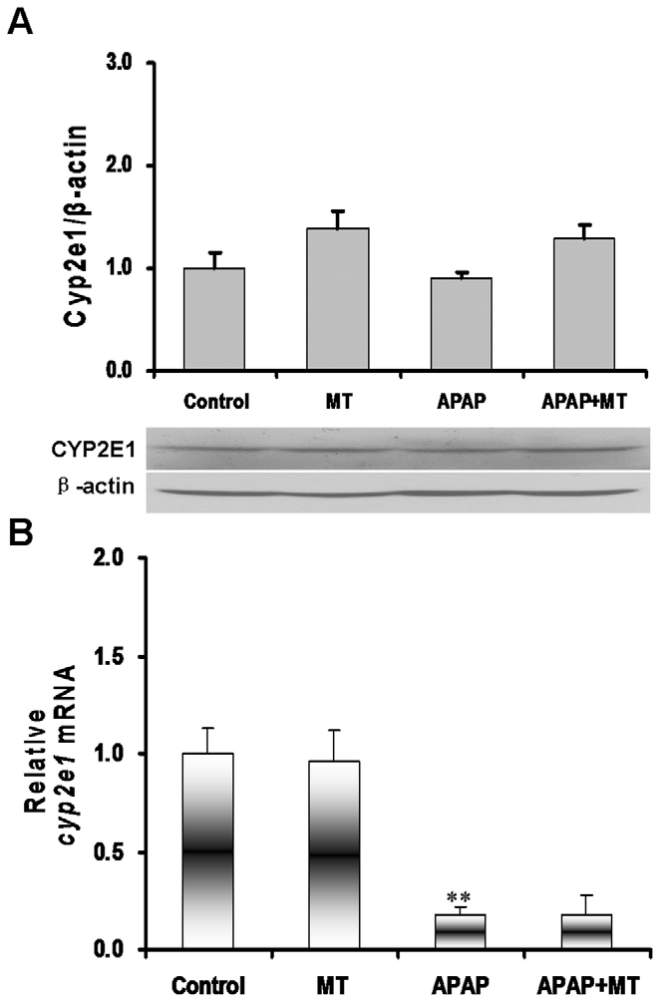
Effects of melatonin on hepatic CYP2E1 expression. Mice were treated as Materials and Methods. Liver samples were collected at 4 h after APAP. (A) Hepatic CYP2E1 was detected by immunoblots. All experiments were repeated for six times. (B) Hepatic *cyp2e1* mRNA was detected using real-time RT-PCR. All data were expressed as means±SEM (n = 6). **P<0.01 as compared with the control.

The effects of melatonin on the expression of hepatic CYP2E1 were analyzed in mice administered with APAP. As shown in Fig. 6A, no significant difference on hepatic CYP2E1 expression was observed among different groups. As shown in Fig. 6B, melatonin alone did not affect the expression of hepatic *cyp2e1* mRNA. Although the level of hepatic *cyp2e1* mRNA was significantly decreased in APAP-treated mice, melatonin had no effect on APAP-induced downregulation of *cyp2e1* mRNA in liver (Fig. 6B).

**Figure 7:**
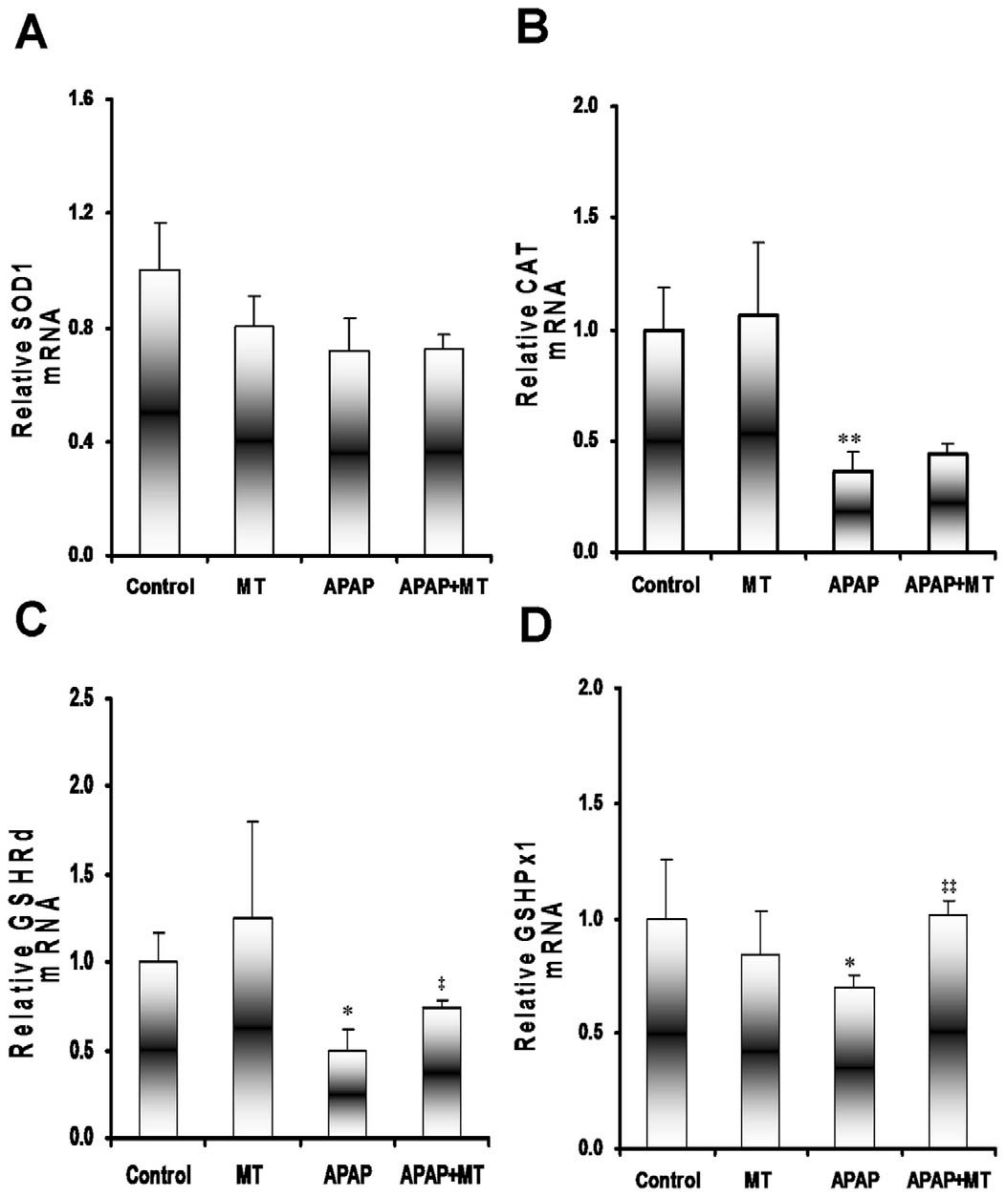
Effects of melatonin on the expression of hepatic antioxidant enzymes. Mice were treated as Materials and Methods. Liver samples were collected at 4 h after APAP. The expression of hepatic antioxidant enzymes were detected using real-time RT-PCR. (A) SOD1; (B) Catalase; (C) GSHRd; (D) GSHPx1. All data were expressed as means ± SEM (n = 6). *P<0.05, **P<0.01 as compared with the control. ‡ *P*<0.05, ‡‡ *P*<0.01 as compared with APAP group.

**Figure 8:**
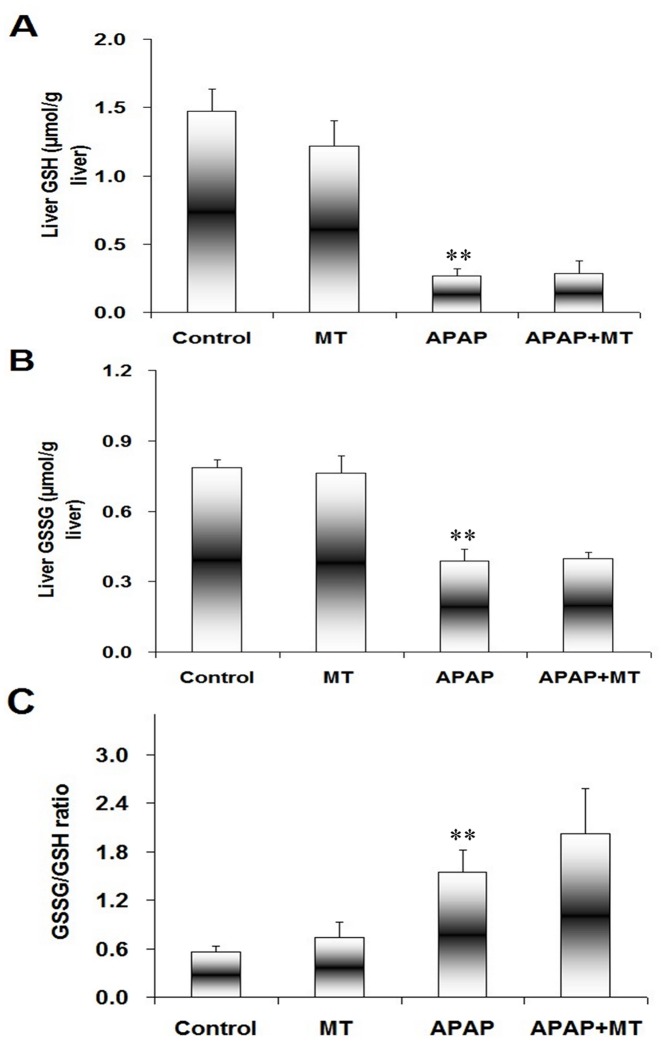
Effects of melatonin on APAP-induced hepatic GSH depletion. Mice were treated as Materials and Methods. Liver samples were collected at 4 h after APAP. Hepatic GSH and GSSG contents were detected. (A) GSH; (B) GSSG; (C) GSSG/GSH. All data were expressed as means ± SEM (n = 6). **P<0.01 as compared with the control.

Finally, the effects of melatonin on the expression of hepatic antioxidant enzymes were analyzed in mice administered with APAP. As expected, melatonin alone had no effect on the expression of hepatic antioxidant enzymes (Fig. 7). Although APAP did not affect the expression of hepatic superoxide dismutase (SOD1, Fig. 7A), the expression of hepatic catalase, GSH reductase (GSH-Rd) and GSH peroxidase (GSH-Px) was downregulated in APAP-treated mice (Figs. 7B–7D). As shown in Figs. 7C and 7D, melatonin significantly attenuated APAP-induced downregulation of hepatic GSH-Rd and GSH-Px. The effects of melatonin on APAP-induced hepatic GSH depletion are presented in Fig. 8. As shown in Fig. 8A, the level of hepatic reduced GSH was obviously decreased 4 h after APAP administration. Although APAP significantly reduced hepatic GSSG content (Fig. 8B), GSSG/GSH ratio was significantly increased in liver of mice treated with APAP (Fig. 8C). Interestingly, melatonin had little effect on APAP-induced GSH depletion.

## Discussion

In the present study, we showed that pretreatment with melatonin significantly alleviated APAP-induced hepatic necrosis and congestion. Correspondingly, melatonin significantly attenuated APAP-induced elevation of serum ALT. In addition, melatonin significantly attenuated APAP-induced increase in hepatic TUNEL+ cells. These results are in agreement with those from two earlier reports [24], [25], in which melatonin protected mice from APAP-induced acute liver failure.

Increasing evidence demonstrates that the sustained activation of JNK plays a major role in APAP-induced hepatotoxicity [6]. Indeed, the present study showed that the level of phosphorylated JNK was significantly increased in liver of mice administered with APAP. Melatonin exerts differential actions on JNK activation in normal and cancer cells. According to an in vitro study, melatonin induces cycle arrest and apoptosis through induction of JNK phosphorylation in HepG2 cell, a hepatocarcinoma cell line [29]. By contrast, two reports from different laboratories showed that melatonin prevented ischemia/reperfusion-induced liver injury through inhibition of toll-like receptor-mediated hepatic JNK activation [30], [31]. In addition, melatonin protected against apoptotic liver damage induced by the rabbit hemorrhagic disease virus through inhibition of hepatic JNK phosphorylation [20]. Consistent with its differential effects on JNK, melatonin also exerts differential actions on cell death in normal and cancer cells [32]. Melatonin increases apoptotic cell death in cancer cells [33]. On the other hand, melatonin exerts its anti-apoptotic effect in normal cells [34]. In the present study, we investigated the effects of melatonin on APAP-induced hepatic JNK activation. Consistent with its anti-apoptotic effect, melatonin inhibited APAP-induced hepatic JNK phosphorylation in a dose-dependent manner. These results suggest that melatonin protects mice from APAP-induced cell death through its inhibition of hepatic JNK phosphorylation.

How melatonin inhibits APAP-induced hepatic JNK phosphorylation remains to be determined. An earlier study demonstrates that RIP1 is upstream of JNK in poly (ADP-ribose) polymerase-1 (PARP-1)-induced mitochondrial dysfunction, nuclear AIF translocation and subsequent cell death [35]. Another report indicates that RIP1-mediated ASK1-interacting protein 1 (AIP1) phosphorylation is essential for the activation of ASK1-JNK/p38 apoptotic signaling [36]. Recently, we found that necrostatin-1, a specific RIP1 inhibitor, significantly alleviated APAP-induced hepatic JNK phosphorylation and caspase-independent cell death (unpublished data). Thus, we guess that RIP1 may be upstream of phosphorylated JNK. Indeed, the present study showed that pretreatment with melatonin significantly attenuated APAP-induced hepatic RIP1 activation. These results suggest that melatonin inhibits APAP-induced hepatic JNK phosphorylation through its inhibition of RIP1 activation.

A recent study indicates that the activated JNK induces Bcl-2 phosphorylation and thereby blocks its translocation to mitochondria [8]. The present study found that the level of mitochondrial Bcl-2 was significantly increased in liver of mice administered with APAP, indicating that Bcl-2 is not downstream target of the activated JNK during APAP-induced acute liver failure. Several studies have demonstrated that the phosphorylated JNK promotes Bax translocation from cytosol to mitochondria through phosphorylation of 14-3-3, a cytoplasmic anchor of Bax [37], [38]. An earlier report observed that the level of hepatic mitochondrial Bax was significantly increased in mice treated with a toxic dose of APAP [39]. Moreover, SP600125, a specific inhibitor of JNK, inhibited APAP-induced mitochondrial Bax translocation [40]. Indeed, the present study found that pretreatment with melatonin obviously attenuated APAP-induced hepatic mitochondrial Bax translocation. These results suggest that Bax may be a potential downstream target of phosphorylated JNK in the process of APAP-induced acute liver failure. Melatonin does not only inhibit APAP-induced hepatic JNK phosphorylation but also subsequent mitochondrial Bax translocation.

The proapoptotic protein Bax translocated to mitochondria can form pores in the outer mitochondrial membrane [41]. Formation of pores in the outer mitochondrial membrane together with formation of the mitochondrial permeability transition (MPT) pores in the inner membrane promotes the early release of AIF, which is thought to trigger DNA cleavage, from the mitochondria to the nuclei. Several reports demonstrate that AIF, which is translocated to the nuclei from the mitochondria, is responsible for the initial DNA fragmentation and subsequent cell death during APAP-induced acute liver failure [3], [4]. The present study showed that nuclear AIF translocation was mainly distributed around hepatic sinus, where nuclei were partially digested. Of interest, pretreatment with melatonin obviously attenuated APAP-induced nuclear AIF translocation. Correspondingly, melatonin significantly alleviated APAP-induced DNA strand breaks around hepatic sinus, as determined by TUNEL assay. These results suggest that melatonin protects mice from nuclear AIF translocation and cell death during APAP-induced acute liver failure.

APAP-mediated acute liver failure is initiated by its reactive metabolite, NAPQI, usually catalysed by hepatic CYP2E1 enzyme [2]. In the present study, we found that the level of hepatic *cyp2e1* mRNA was significantly decreased when mice were administered with APAP. These results are in agreement with others, in which the expression of hepatic CYP2E1 protein was significantly decreased after repeat exposure to incremental doses of APAP. Interestingly, the present study showed that no significant downregulation of hepatic CYP2E1 protein was observed at 4 h after APAP treatment. Indeed, an earlier report also showed no significant difference on the expression of hepatic CYP2E1 protein 6 h after APAP treatment, whereas there was a significant decrease in hepatic CYP2E1 protein 24 h following APAP treatment of rats. A recent study showed that melatonin significantly inhibited the expression of *cyp2e1* mRNA in nigrostriatal tissues [42]. The present study investigated the effects of melatonin on the expression of hepatic CYP2E1 in mice treated with APAP. We found that a single dose of melatonin did not affect the expression of hepatic CYP2E1 protein. These results suggest that melatonin protects against APAP-induced acute liver failure probably downstream of CYP2E1.

Numerous reports have demonstrated that hepatic GSH depletion is involved in the process of APAP-mediated sustained activation of JNK and subsequent acute liver injury [5], [43]. The present study showed that hepatic GSH content was significantly decreased in mice treated with APAP. By contrast, hepatic GSSG/GSH ratio was significantly increased in mice treated with APAP. Several reports found that melatonin significantly alleviated hepatic GSH depletion [44], [45]. Indeed, the present study showed that APAP-induced downregulation of hepatic GSHRd and GSHPx1 was partially reversed by melatonin. Thus, we investigated the effects of melatonin on APAP-induced hepatic GSH depletion. Unexpectedly, melatonin had little effect on APAP-induced hepatic GSH depletion. These results suggest that melatonin inhibits APAP-induced sustained JNK activation independent of its antioxidant effect.

In summary, the present study indicates that melatonin protects against AIF-dependent cell death during APAP-induced acute liver failure. Although it has little effect on APAP-evoked hepatic GSH depletion, melatonin is able to inhibit APAP-induced hepatic RIP1 activation, reducing both JNK phosphorylation and mitochondrial translocation of Bax. Correspondingly, melatonin inhibits APAP-induced AIF translocation from the mitochondria to the nuclei, which seems to be responsible for the significant reduction on APAP-induced cell death when melatonin is administered. Thus, melatonin may have potential preventive and therapeutic utilities for protecting against APAP-induced acute liver failure.
